# Optimization of Early Mobilization Program for Patients With Acute Ischemic Stroke: An Orthogonal Design

**DOI:** 10.3389/fneur.2021.645811

**Published:** 2021-04-12

**Authors:** Min Zhang, Qing Wang, Yuanyuan Jiang, Huiling Shi, Tiantian Peng, Mengyue Wang

**Affiliations:** ^1^Department of Neurology, Nanjing Drum Tower Hospital, Nanjing University Medical School, Nanjing, China; ^2^Department of Nursing, Nanjing Drum Tower Hospital, Nanjing University Medical School, Nanjing, China

**Keywords:** acute ischemic stroke, early mobilization, orthogonal design, early rehabilitation, program optimization

## Abstract

**Background and purpose:** Early mobilization is considered to have favorable outcomes for stroke patients, but there is currently a lack of specific data to guide this early mobilization, including the initiation time, intensity, frequency, and duration of each activity. Therefore, the optimal strategy for early mobilization is unclear. In this study, we investigated the best combination of different factors to achieve early mobilization, to develop the optimal program.

**Methods:** We conducted an L_9_ (3^3^) orthogonal experiment with a blinded follow-up assessment. Patients with ischemic stroke, admitted to a stroke unit within 24–72 h of its onset, were recruited. Eligible subjects were randomly assigned to one of nine different programs of early mobilization. The outcomes were assessed at baseline, discharge, and 1 and 3 months after discharge to observe the changes in various efficacy indicators and determine the main factors affecting outcome.

**Results:** We analyzed 57 of 63 patients, after six were excluded for poor compliance, failure to cooperate with the study, or worsening of the disease. The initiation time, intensity, and frequency of mobilization were the main factors affecting outcome (all *P* < 0.05), and the duration of each activity was a secondary factor (*P* > 0.05). A comprehensive analysis of the various parameters showed that the optimal level of the early mobilization program was an initiation time: 24–48 h after stroke; intensity: bed and chair transfer, sitting out of bed, standing and walking or climbing stairs when mobility permitted; frequency: 2–3 times/day; duration of each mobilization: determined according to the actual situation of the patient.

**Conclusions:** Early rehabilitation with high-intensity physical exercise at 24–48 h after the onset of stroke, 2–3 times/day, may benefit stroke patients. Applying the optimized program of early mobilization to stroke patients effectively alleviated their symptoms of neurological deficit, improved their capacity for self-care, restored their self-efficacy, improved their quality of life and social participation, and reduced post-stroke fatigue at 3 months.

## Introduction

Ischemic stroke confers varying degrees of neurological deficit and lasting functional limitations on most patients, which have serious social and economic consequences, and are a potential public-health problem of global concern (1–3). Early mobilization is one of the core concepts of the early rehabilitation of these patients, and can prevent or reduce inactivity-related complications, promote neurological recovery, and improve patient outcomes (4–6). Researchers reported early on the benefits of early mobilization for stroke patients in Norway in 1999 (7). The activities involved include bed and chair transfer, sitting out of bed, standing, and walking. Although current guidelines recommend leaving bed “early” during the acute phase after stroke and the time window has been shortened from the original 72 h to 24 h, these guidelines do not specify how or if early exercise optimizes patient outcomes (8, 9). Many published studies have shown that the effectiveness and safety of early mobilization after acute stroke are inconsistent. In a series of studies, A Very Early Rehabilitation Trial (AVERT), the author did not recommend any specific time for the initiation of rehabilitation, whereas the studies showed that mobilization within 24 h of stroke may have adverse consequences (10, 11). However, the multicenter Early Sitting in Ischemic Stroke Patients (SEVEL) trial found that step-by-step sitting exercises within 24 h of stroke could improve the patient's neurological deficit at the time of discharge and his/her capacity for daily living within 3 months of stroke (12). A recent meta-analysis concluded that insufficient evidence supports the notion that early mobilization improves patients' neurological deficits and disabilities, and that its efficacy remains to be established (13). Although researchers have begun to consider early mobilization, the optimal time to commence it is still unknown, and few studies have focused on the optimal frequency and intensity of mobilization. Bernhardt and his team conducted a dose–effect analysis of early activities in a multicenter, large-sample study (14). Their results showed that short-term and high-frequency activities within 24 h of stroke were more likely to improve the patient outcomes than other measures, but prescribed no quantified activities. The latest guidelines from the American Stroke Association (8) indicate that high-frequency activity within 24 h of the onset of stroke reduces the likelihood of a favorable outcome at 3 months. The optimal dose is unclear.

The traditional method of evaluating such a multicomponent program, which combines different levels of activity, initiation times, frequencies, and intensities, is with a parallel-group randomized trial design, with program revisions and subsequent randomized trials based on the results. A particular disadvantage of this method is that it cannot test the influence of a single component or the interaction between the components of a multicomponent processing program. A large number of samples and many experiments are required. Therefore, in this study, we used an orthogonal design to resolve this problem. The orthogonal design method efficiently deals with multifactor design and screens for optimal levels with an orthogonal design table (15).

The multiphase optimization strategy (MOST) has been described as an alternative framework for optimizing multicomponent interventions with a rigorous, multistage strategy (16). From a MOST perspective, optimization begins with a screening phase, during which the most promising treatment components are identified and grounded within a theoretical framework. The second stage is used to refine the doses of the components and their combinations, and to explore differences in the mitigating effects based on the participant characteristics. The final phase includes a confirmation of the benefits of the optimized intervention, usually with a randomized trial design (17, 18).

**Figure 1:**
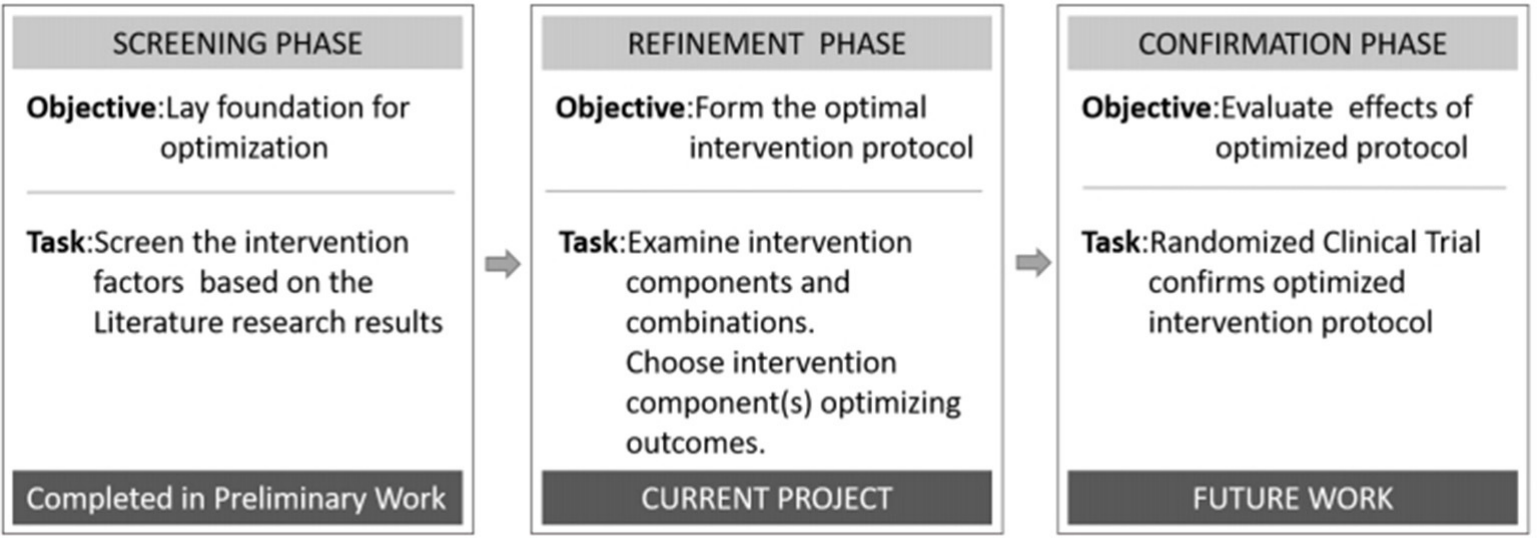
Phases of the multiphase optimization strategy (MOST) used to develop a program to optimize early post-stroke mobilization.

In this study, we used MOST as the theoretical framework to refine each phase (Figure 1). Related literature studies of the early stage of post-stroke mobilization confirmed the significance of early activities, but there is no quantitative activity guidance plan. Therefore, in this study, we used an orthogonal design to evaluate the main effects of four factors (initiation time, frequency, intensity, and time per activity) and their interactive effects with the goal of developing an optimized program, which can be evaluated in future randomized trials. Therefore, the overall goal of this project was to define an optimized program for the early activities of patients with acute ischemic stroke in order to promote the effective implementation of early activities and ensure their safety.

## Methods

### Study Design and Setting

This study had an orthogonal design. The subjects of the study were patients admitted to the Stroke Unit of the Department of Neurology, Drum Tower Hospital Affiliated to Nanjing University Medical School with acute ischemic stroke between October 2019 and June 2020. The institutional Ethics Committee approved the study. Each patient signed an informed consent form.

### Participants

During the recruitment period, the principal researcher screened all patients admitted to the study according to the following criteria.

Inclusion criteria: (1) patients were aged 18–80 years and diagnosed with acute ischemic stroke; (2) neurological deficit, according to the National Institutes of Health Stroke Scale (NIHSS; score of 1 ≤ NIHSS < 16); (3) deficiency in the activities of daily living (Barthel Index ≤ 85); (4) ability to answer questions correctly, Modified Early Warning Score (MEWS) ≤ 2, and a single-dimensional score < 2; and (5) informed consent was obtained from the patient or his/her guardian before the study.

Exclusion criteria: (1) mental illness; (2) other systemic comorbidities, limb fractures, palliative care, or advanced malignant tumor; (3) admitted to hospital > 72 h after onset; or (4) enrollment in another intervention trial.

Elimination criteria: (1) neurological deficit symptoms gradually worsening within 2 h of admission; (2) poor compliance and non-cooperation with the study; or (3) severe adverse events, complications, or specific physiological changes precluded continuation, or the patient quit voluntarily.

### Grouping

According to a preliminary literature search, the patients with acute ischemic stroke were screened for the parameters of early mobilization, including four factors and three levels (Table 1).

**Table 1:** Factors and levels used in the orthogonal experiment.

**Level**	**Factors**			
	**A: Initiation time (h)**	**B: Intensity**	**C: Frequency (times/d)**	**D: Duration time of each mobilization (min)**
1	<24	Bed and chair transfer + sitting out of bed	1	<30
2	24–48	Bed and chair transfer + sitting out of bed+standing	2–3	30–60
3	>48	Bed and chair transfer + sitting out of bed+standing+walking (or climbing stairs)^*^	>3	>60

Instructions:

* *If the patient's muscle strength was less than level 3, the maximum activity that could be completed within the range of physical functions was used as the activity intensity B_3_, including weight-bearing training of the lower limbs under professional guidance and equipment-assisted walking training*.

Some patients were admitted to the hospital more than 24 h after the onset of stroke, according to the guidelines (19), although it is recommended that out-of-bed activities be implemented within 24 h of admission. Therefore, the time of initiation of mobilization could not be included in the orthogonal design as an influencing factor and an L_9_ (3^3^) orthogonal design was used in this study (Table 2). Because previous studies (12, 14) have shown that early activities have a good effect on the outcomes of patients, we included no control group without mobilization in this study.

**Table 2:** L_9_ (3^3^) orthogonal design.

**Program number**	**Column number**		
	**B**	**C**	**D**
1	1	1	1
2	2	2	2
3	3	3	3
4	1	2	3
5	2	3	1
6	3	1	2
7	1	3	2
8	2	1	3
9	3	2	1

In Table 1, A, B, C, and D denote the different factors, and 1, 2, and 3 indicate the three different levels of each factor. The nine specific kinds of implementation scheme are shown in Table 2.

### Study Measures

Before the patient was enrolled in the study, the researcher would interpret the study to the patient and caregivers, including the content of the activity plan to be followed during the study, and then the patient himself/herself would sign the informed consent.

All the enrolled patients were randomly assigned to one of the nine different programs with a computer-generated randomization procedure using opaque envelopes. However, the participants in this study could not be blinded to treatment because our purpose was to optimize the program, rather than to evaluate the efficacy of early mobilization. The therapists and nurses who provided guidance on the early activities could not be blinded. However, the follow-up assessments at discharge and 1 and 3 months after discharge were performed by a researcher blinded to the participants' groups.

**Figure 2:**
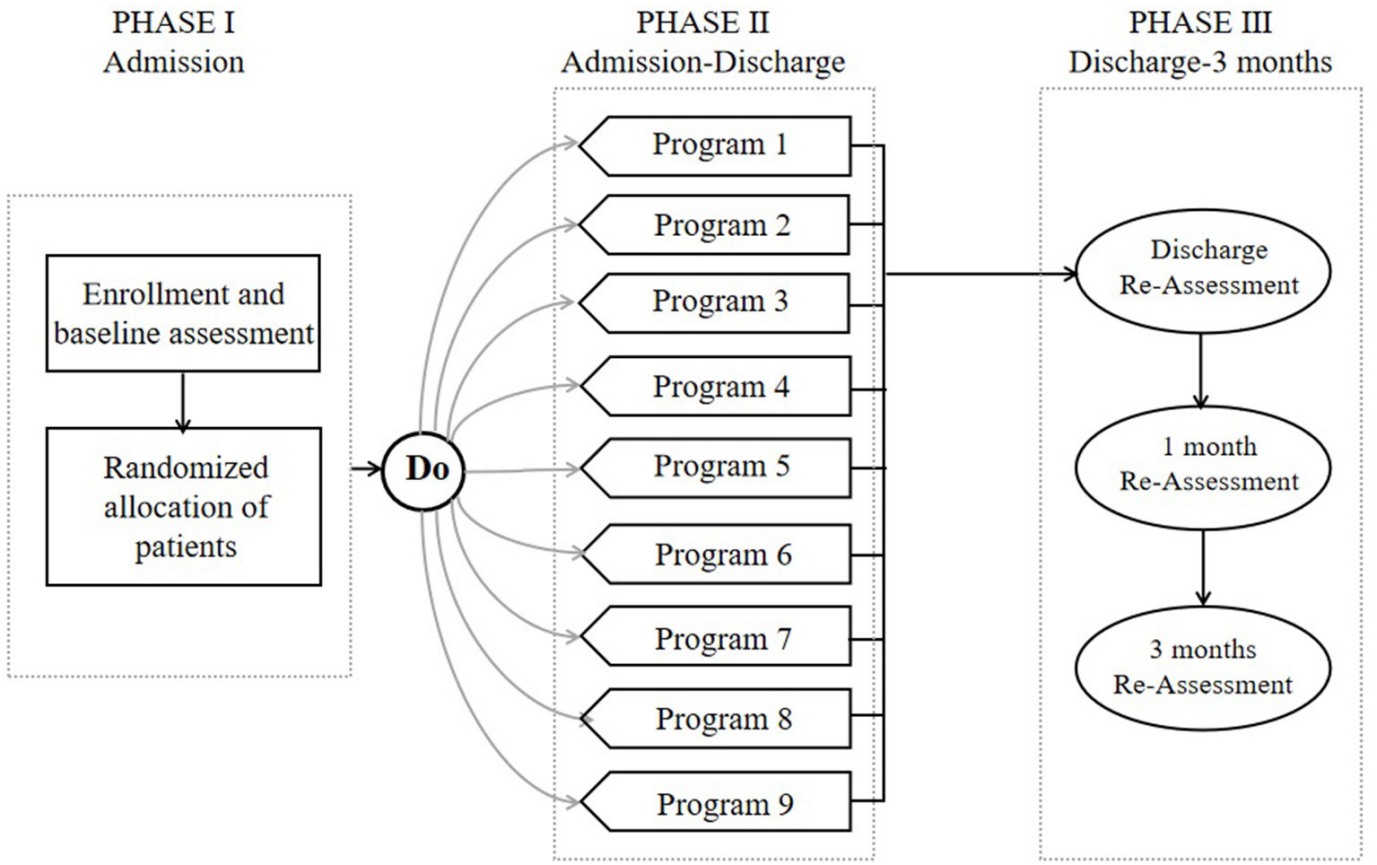
Study design showing the three phases and four assessments.

Assessments were made at baseline, discharge, and 1 and 3 months after discharge. The baseline characteristics of the subjects were collected at the beginning of the study, and included age, sex, job, salary, stroke side, severity, and risk factors (hypertension, diabetes mellitus, ischemic heart disease, hypercholesterolemia, smoking, atrial fibrillation, previous stroke, and transient ischemic attack). Premorbid disability, the Rankin Scale admission score, the time to first mobilization after symptom onset, and the caregiver were recorded. The short-term effects of different early mobilization programs were evaluated at an examination at the time of patient discharge. The persistence of these effects was evaluated at the 1- and 3-month assessments. The research design is outlined in Figure 2.

### Early Mobilization

Out-of-bed mobilization included sitting, standing, and walking, which were performed with or without assistance, as described in the AVERT program (20). All the mobilization programs were implemented based on the orthogonal experimental design and were delivered by professional therapists or nurses. The patient's body temperature, blood pressure, respiration, heart rate, blood oxygen saturation, and level of consciousness were evaluated before mobilization, and the patient only exercised when his/her condition permitted. The frequency, dose, and intensity of activities were recorded in detail by the therapists or nurses. These records also included whether the patient suffered headache, dizziness, shortness of breath, or other uncomfortable symptoms during mobilization; whether there were any activity-related adverse events, such as falls; and whether there were any deep vein complications, such as blood clots, pressure sores, or constipation. Patients wore sports bracelets while moving, which recorded in detail the exercise start time, duration, and number of steps taken while walking.

Dose was monitored by a specifically assigned staff member to ensure good compliance with the study. The patient continued to undertake the prescribed exercises every day during his/her hospital stay until discharge.

### Outcome Assessment

To assess the effectiveness of the study, after the patients were enrolled, they were evaluated until 3 months after discharge. Table 3 summarizes additional outcome measures. Demographic data, stroke information, and medical history were extracted from the participants' hospital charts. The participants completed a questionnaire at baseline, which asked about their social support, employment, living situation, and so on, which are factors that could influence their compliance with early post-stroke activities. Each patient was scored on the NIHSS at enrolment in and discharge from the study. Some clinical assessments were scored by a blinded research assistant at four time points: (1) study enrolment; (2) discharge; (3) 1 month after discharge; and (4) 3 months after discharge. These primary outcomes were measured with the following tests: the modified Rankin Scale (mRS), the Barthel Index (BI); the Fatigue Severity Scale (FSS); and the Irritability Depression and Anxiety Scale (IDA). Some other clinical assessments reflected the patient's rehabilitation efficacy, quality of life, and social participation after discharge, so these primary outcomes were measured at 1 and 3 months after discharge. The tests used included: the Stroke Self-Efficacy Questionnaire (SSEQ); the Stroke-Specific Quality of Life Scale (SS-QoL); and the Impact on Participation and Autonomy Questionnaire (IPA). The participants were asked to report falls (an event that resulted in the person coming to rest unintentionally on the ground or at another lower level) related to these activities and other complications related to inactivity, such as pneumonia, deep vein thrombosis, and pressure ulcers, within the 3 months after discharge. And these were secondary outcomes.

**Table 3:** Cohort descriptors and outcome measures.

	**Study enrolment**	**Discharge**	**1 month post-discharge**	**3 months post-discharge**	**During 3-month follow-up**
Demographics	✓				
Time post-stroke	✓				
Lesion location	✓				
Medical history	✓				
Risk factors for stroke	✓				
Medications	✓				
Changes in health/medications		✓	✓	✓	
NIHSS	✓	✓			
mRS	✓	✓	✓	✓	
BI	✓	✓	✓	✓	
FSS	✓	✓	✓	✓	
IDA	✓	✓	✓	✓	
SS-QoL			✓	✓	
IPA			✓	✓	
SSEQ			✓	✓	
Falls in daily life					✓^*^
Other complications					✓^*^

* *Data collected repeatedly during the 3 months follow-up period*.

*NIHSS, National Institutes of Health Stroke Scale; mRS, modified Rankin Scale; BI, Barthel Index; FSS, Fatigue Severity Scale; IDA, Irritability Depression and Anxiety Scale; SS-QoL,Stroke-Specific Quality of Life Scale; IPA, Impact on Participation and Autonomy Questionnaire; SSEQ,Stroke Self-Efficacy Questionnaire*.

The NIHSS was used to assess the degree of neurological impairment in patients and to understand the recovery of the disease. The lower the score, the better the neurological function (21). The outcome was defined as favorable with an mRS score of 0–2 (no or minimum disability), whereas a poor outcome was defined as an mRS score of 3–6 (moderate or severe disability, or death) (22). The BI was used to determine the functional status of the examined patients based on specific daily activities (23). Previously reported intraclass correlation coefficient (ICC) for the BI, representing inter-rater reliability, was 0.99 (24). The FSS was used to evaluate patients' post-stroke fatigue (25). Choi-Kwon et al. in Korea used FSS to evaluate post-stroke fatigue, and the Cronbach's α was 0.928, indicating good internal consistency (26). The IDA was used to evaluate the negative emotions of the patients, which was compiled by Snaith et al. (27), Cronbach's α for each dimension of the scale was 0.419–0.769 (28). Higher SSEQ scores indicated better rehabilitation and self-efficacy. Cronbach's α for each dimension of the scale was 0.969 (29). The higher the SS-QOL scale score, the better the quality of life (30). The IPA was compiled by the Dutch scholar (31), and the Chinese scholar (32) has finished and revised it. The higher the score, the lower the level of social participation. Cronbach's α is 0.782–0.965 (32).

### Data Collection Method and Follow-Up

The nurse responsible recorded the patient's activities daily and collated the monitoring data recorded on the patient's bracelet. We used a computer to establish a follow-up system, create relevant evaluation forms, and send them to the patient's mobile phone to be completed at the specified time. We used questionnaires for on-site assessments when the patients were admitted and discharged. The questionnaires used at 1 and 3 months after discharge from hospital were conveyed to the mobile terminal of the patient or caregiver, to be completed within 1 week. If the questionnaires were not submitted within 1 week or were incomplete, we followed-up the patient by telephone. All patients were followed-up for 3 months to assess the sustainability and feasibility of the research effects.

### Sample Size Estimation

The sample size was estimated based on the orthogonal design of an animal experiment model (33), in which each group of experiments was repeated six times, and a good result was obtained. Assuming a 20% shedding rate, it was decided to repeat each group of experiments seven times, and a total of 63 patients were included. These 63 patients included were randomly divided into nine groups with computer-generated randomization, with seven patients in each group.

### Statistical Analysis

The SPSS 19.0 software (IBM, Armonk, NY, USA, 2012) was used for all data processing and analysis. Normally distributed data are presented as means ± standard deviations and these data were compared with one-way analysis of variance (ANOVA). Non-normally distributed data are presented as medians and compared with the Kruskal–Wallis *H* test. Categorical variables were compared with a χ^2^ test. Because the mobilization initiation time (factor A) could not be included in the orthogonal design table as an initial influencing factor, because it might have affected the patient's outcome, it was classified at three levels and included in the final statistics, and multifactor ANOVA was used to determine the statistical significance. *P* < 0.05 was considered significant. The range analysis method was used to determine the major and minor orders between the processing parameters, to obtain the optimum combination of levels.

## Results

### Baseline Characteristics

The 63 patients included were randomly divided into nine groups with seven patients in each group; 57 patients finished the training and follow-up assessment, whereas four patients did not cooperate with the program, and two patients were excluded due to disease worsening. No patients died in the process of research. Table 4 presents the baseline characteristics of the patients. There were no differences among the groups in terms of age, sex, hospital stay, stroke severity, BI, FSS, IDA scores, or other risk factors (all *P* > 0.05). Stroke severity at admission was evaluated with NIHSS.

**Table 4:** Baseline characteristics of the patients.

	**Program1** **(*n* = 7)**	**Program2** **(*n* = 6)**	**Program3** **(*n* = 6)**	**Program4** **(*n* = 6)**	**Program5** **(*n* = 6)**	**Program6** **(*n* = 7)**	**Program7** **(*n* = 6)**	**Program8** **(*n* = 6)**	**Program9** **(*n* = 7)**	***P***
Age (years)	64.00 ± 7.98	65.67 ± 6.41	67.50 ± 7.26	57.67 ± 8.85	62.00 ± 12.02	56.00 ± 13.10	64.00 ± 14.67	71.00 ± 9.42	62.00 ± 8.83	0.262
Sex (male)	5 (71.4%)	5 (83.3%)	4 (66.7%)	6 (100%)	4 (66.7%)	4 (57.1%)	2 (33.3%)	4 (66.7%)	2 (28.6%)	0.222
Hospital stay(days)	11.57 ± 2.88	11.50 ± 2.07	10.17 ± 3.82	10.83 ± 0.75	11.50 ± 1.87	13.71 ± 5.82	11.00 ± 4.00	14.50 ± 7.87	11.14 ± 3.02	0.661
NIHSS score	3.00 (2.00,3.00)	2.00 (1.00,4.00)	2.50 (1.75,4.00)	1.50 (1.00,3.00)	4.50 (1.75,9.25)	3.00 (1.00,5.00)	7.00 (2.50,10.50)	6.50 (3.75,7.25)	4.00 (2.00,7.00)	0.053
BI score	58.57 ± 17.01	50.83 ± 12.01	64.17 ± 7.36	70.00 ± 8.37	45.83 ± 16.56	65.71 ± 14.56	54.17 ± 17.44	44.17 ± 27.28	61.43 ± 13.76	0.068
FSS score	32.00 ± 14.63	25.00 ± 14.68	28.67 ± 19.49	27.67 ± 13.52	32.33 ± 11.18	34.00 ± 19.65	34.83 ± 15.01	33.83 ± 17.92	30.29 ± 15.19	0.975
IDA score	26.29 ± 11.19	19.67 ± 7.28	26.67 ± 12.80	21.50 ± 11.26	26.17 ± 11.37	16.86 ± 12.24	21.00 ± 15.45	28.33 ± 12.36	27.71 ± 18.01	0.721
**Risk Factors**										
Hypertension	6 (85.7%)	4 (66.7%)	5 (83.3%)	3 (50%)	4 (66.7%)	6 (85.7%)	5 (83.3%)	5 (83.3%)	5 (71.4%)	0.852
Diabetes mellitus	0 (0%)	2 (33.3%)	4 (66.7%)	1 (16.7%)	3 (50%)	2 (28.6%)	2 (33.3%)	2 (33.3%)	2 (28.6%)	0.405
Hypercholesterolemia	1 (14.3%)	1 (16.7%)	0 (0%)	1 (16.7%)	0 (0%)	1 (14.3%)	0 (0%)	3 (50%)	0 (0%)	0.166
Previous stroke or TIA	3 (42.9%)	3 (50%)	2 (33.3%)	0 (0%)	0 (0%)	2 (28.6%)	2 (33.3%)	1 (16.7%)	1 (14.3%)	0.393
Smoking	0 (0%)	2 (33.3%)	1 (16.7%)	2 (33.3%)	1 (16.7%)	1 (14.3%)	0 (0%)	0 (0%)	0 (0%)	0.358
Ischemic heart disease or Atrial fibrillation	1 (14.3%)	0 (0%)	0 (0%)	0 (0%)	0 (0%)	0 (0%)	0 (0%)	0 (0%)	0 (0%)	0.529

*NIHSS, the National Institutes of Health Stroke Scale; BI, the Barthel Index; FSS, Fatigue Severity Scale; IDA, Irritability Depression and Anxiety Scale*.

### Range Analysis

In the range analysis method, the range (*R* value) of each factor is calculated because this reflects the influence of each factor at a specific level on the reaction system. A larger K value indicates a better reaction level. The calculation results are listed in Table 5. For example, as shown in Table 5, for the indicator BI at 3 months, the order of influence of four factors affecting early mobilization was frequency > intensity > duration of each activity >initiation time. The excellent combination of the early mobilization program was C_2_B_3_D_1_A_2_ (frequency: 2–3 times/day; intensity: bed and chair transfer + sitting out of bed + standing + walking (or climbing stairs); duration of each activity: <30 min; initiation time: 24–48 h). However, because many indicators were examined, the order of influence of four factors on the early activities was inconsistent for the various indicators observed, so this range analysis only determined the optimal level of a certain factor's impact on the effect, and not the final significance of each influencing factor.

**Table 5:** Results of the range analysis of the effect of early mobilization.

		**At discharge**				**1 month post-discharge**				**3 months post-discharge**			
		**A**	**B**	**C**	**D**	**A**	**B**	**C**	**D**	**A**	**B**	**C**	**D**
BI	K1	70.714	72.937	72.698	71.706	88.016	87.460	88.690	86.389	91.151	92.262	92.579	93.810
	K2	76.151	62.500	76.825	73.333	89.841	79.444	92.817	87.222	92.817	84.722	93.770	90.833
	K3	69.048	80.476	66.389	70.873	81.984	92.937	78.333	86.230	86.548	93.532	84.167	85.873
	R	7.103	17.976	10.436	2.460	7.857	13.493	14.484	0.992	6.269	8.810	9.603	7.937
	Factor priority	B>C>A>D				C>B>A>D				C>B>D>A			
	Excellent combination	B_3_C_2_A_2_D_2_				C_2_B_3_A_2_D_2_				C_2_B_3_D_1_A_2_			
mRS	K1	2.746	2.635	2.683	2.635	2.214	2.214	2.143	2.230	2.071	1.794	1.937	1.873
	K2	2.548	3.167	2.333	2.778	1.929	2.667	1.849	2.333	1.643	2.444	1.690	2.000
	K3	2.833	2.325	3.111	2.714	2.516	1.778	2.667	2.095	2.246	1.722	2.333	2.087
	R	0.285	0.842	0.778	0.143	0.587	0.889	0.818	0.238	0.603	0.722	0.643	0.214
	Factor priority	B>C>A>D				B>C>A>D				B>C>A>D			
	Excellent combination	B_3_C_2_A_2_D_1_				B_3_C_2_A_2_D_3_				B_3_C_2_A_2_D_1_			
FSS	K1	30.024	32.135	29.032	30.016	27.921	23.865	23.341	25.405	23.603	19.825	19.556	20.079
	K2	23.563	31.056	28.270	26.167	17.198	29.111	23.873	24.833	14.675	24.167	21.698	20.222
	K3	33.048	23.444	29.333	30.452	29.984	22.127	27.889	24.865	24.643	18.929	21.667	22.619
	R	9.485	8.691	1.063	4.285	12.786	6.984	4.548	0.572	9.968	5.238	2.142	2.540
	Factor priority	A>B>D>C				A>B>C>D				A>B>D>C			
	Excellent combination	A_2_B_3_D_2_C_2_				A_2_B_3_C_1_D_2_				A_2_B_3_D_1_C_1_			
IDA	K1	10.937	11.881	11.683	11.246	10.103	10.103	9.825	10.024	10.444	9.111	9.913	7.857
	K2	11.635	12.111	12.143	13.111	8.056	11.000	8.754	10.667	7.190	10.889	8.024	11.000
	K3	13.032	11.611	11.778	11.246	11.754	8.810	11.333	9.222	11.690	9.325	11.389	10.468
	R	2.095	0.500	0.460	1.865	3.698	2.190	2.579	1.445	4.500	1.778	3.365	2.611
	Factor priority	A>D>B>C				A>C>B>D				A>C>D>B			
	Excellent combination	A_1_D_1(or3)_B_3_C_1_				A_2_C_2_B_3_D_3_				A_2_C_2_D_1_B_1_			
SSEQ	K1					94.770	97.103	94.722	97.460	100.770	103.825	100.079	109.024
	K2					103.008	83.389	109.468	85.833	109.587	94.611	112.643	98.333
	K3					84.135	101.421	77.722	98.619	92.198	104.119	89.833	95.198
	R					18.873	18.032	31.746	12.786	17.389	9.508	22.810	13.826
	Factor priority					C>A>B>D				C>A>D>B			
	Excellent combination					C_2_A_2_B_3_D_3_				C_2_A_2_D_1_B_3_			
SS-QoL	K1					203.286	201.175	199.913	201.270	204.357	210.024	209.579	214.952
	K2					209.349	185.444	219.484	196.444	218.500	193.222	219.095	207.222
	K3					187.151	213.167	180.389	202.071	198.484	218.095	192.667	199.167
	R					22.198	27.723	39.095	5.627	20.016	24.873	26.428	15.785
	Factor priority					C>B>A>D				C>B>A>D			
	Excellent combination					C_2_B_3_A_2_D_1_				C_2_B_3_A_2_D_1_			
IPA	K1					37.429	33.040	34.365	33.421	31.071	27.183	25.905	24.944
	K2					28.325	49.444	24.992	40.611	21.333	40.000	21.540	28.500
	K3					44.214	27.484	50.611	35.937	36.984	22.206	41.944	35.944
	R					15.889	21.960	25.619	7.190	15.651	17.794	20.404	11.000
	Factor priority					C>B>A>D				C>B>A>D			
	Excellent combination					C_2_B_3_A_2_D_1_				C_2_B_3_A_2_D_1_			

*A, Initiation time; B, intensity; C, frequency; D, duration of each activity*.

*Ki (1, 2, 3) was obtained by adding any number of columns corresponding to factor i. R is the difference between the maximum value and the minimum value of Ki (1, 2, 3) in any column*.

### Multivariate ANOVA

Multivariate ANOVA was used to determine the impacts of the different factors. According to mathematical statistics, the *P*-value is considered to be the probability of obtaining a certain value. When *P* < 0.05, a factor was considered to have a significant effect on the experimental results. Tables 6, **9**–**11** show that for the 1 and 3 months outcomes, the probabilities of B and C were lower than 0.05 for indicators BI, SSEQ, SS-QoL, and IPA, with p(C) < p(B). This indicates that factor C (frequency) had the stronger effect on these indicators, followed by factor B (intensity). The results of ANOVA are consistent with the results of the range analysis shown in Table 5, which shows that factor C (frequency) was the most influential factor among these indicators. This indicates that the selected levels in the orthogonal design plan are reasonable and that the error is also reasonable at a certain level. However, for indicator mRS shown in Table 12, p(B) < p(C), which indicates that factor B (intensity) had a stronger effect on mRS than factor C (frequency). Despite this, combining these results with the range analysis results shown in Table 5, we still concluded that the optimal level of frequency and intensity was C_2_B_3_ [frequency: 2–3 times/day; intensity: bed and chair transfer + sitting out of bed + standing + walking (or climbing stairs)].

**Table 6:** ANOVA of the factors affecting the Barthel Index.

**BI**	**Factors**	**DF**	**MS**	***F*-value**	***p*-value**	**Significance**
At discharge	A	2	260.784	0.932	0.401	ns
	B	2	1533.145	5.480	0.007^**^	
	C	2	508.104	1.816	0.174	ns
	D	2	28.773	0.103	0.902	ns
At 1-month discharge	A	2	319.563	1.913	0.159	ns
	B	2	865.602	5.181	0.009^**^	
	C	2	1022.884	6.123	0.004^**^	
	D	2	5.220	0.031	0.969	ns
At 3-month discharge	A	2	199.325	1.305	0.281	ns
	B	2	420.262	2.751	0.074	ns
	C	2	504.015	3.300	0.045^*^	
	D	2	152.741	2.030	0.142	ns

BI, Barthel Index; DF, degree of freedom; MS, mean square;

* significant, 0.01 < p < 0.05;

** very significant, 0.001 < p < 0.01;

*** highly significant, p < 0.001; non-significance is indicated as ns (p > 0.05).

Factor A (initiation time) only affected FSS and SS-QoL at 1 month after discharge as shown in Tables 7, **10**, However, similarly, when combined with the range analysis results in Table 5, we inferred that the optimal level of initiation time was A_2_ (initiation time: 24–48 h). None of the four factors were shown to affect IDA in Table 8, and the significance of factor D (duration of each activity) was unclear (all *p* > 0.05) from Tables 6–11.

**Table 7:** ANOVA of the factors affecting the severity of fatigue.

**FSS**	**Factors**	**DF**	**MS**	***F*-value**	***p*-value**	**Significance**
At discharge	A	2	443.608	3.293	0.046^*^	
	B	2	436.123	3.237	0.048^*^	
	C	2	5.596	0.042	0.959	ns
	D	2	102.351	0.760	0.473	ns
At 1-month discharge	A	2	890.500	5.916	0.005^**^	
	B	2	246.204	1.636	0.205	ns
	C	2	114.436	0.760	0.473	ns
	D	2	1.997	0.013	0.987	ns
At 3-month discharge	A	2	567.524	4.134	0.022^*^	
	B	2	145.609	1.061	0.354	ns
	C	2	29.254	0.213	0.809	ns
	D	2	38.621	0.281	0.756	ns

FSS, Fatigue Severity Scale; DF, degree of freedom; MS, mean square;

* significant, 0.01 < p <0.05;

** very significant, 0.001 < p <0.01;

*** highly significant, p < 0.001; non-significance is indicated as ns (p > 0.05).

**Table 8:** ANOVA of the factors affecting the negative emotions.

**IDA**	**Factors**	**DF**	**MS**	***F*-value**	***p*-value**	**Significance**
At discharge	A	2	21.511	0.653	0.525	ns
	B	2	1.188	0.036	0.965	ns
	C	2	1.129	0.034	0.966	ns
	D	2	21.385	0.649	0.527	ns
At 1-month discharge	A	2	64.878	2.161	0.126	ns
	B	2	23.076	0.769	0.469	ns
	C	2	30.895	1.029	0.365	ns
	D	2	9.679	0.322	0.726	ns
At 3-month discharge	A	2	102.031	2.317	0.110	ns
	B	2	17.324	0.393	0.667	ns
	C	2	52.595	1.194	0.312	ns
	D	2	54.556	1.239	0.299	ns

*IDA, Irritability Depression and Anxiety Scale; DF, degree of freedom; MS, mean square; non-significance is indicated as ns (p > 0.05)*.

**Table 9:** ANOVA of the factors affecting self-efficacy.

**SSEQ**	**Factors**	**DF**	**MS**	***F*-value**	***p*-value**	**Significance**
At 1-month discharge	A	2	1692.049	2.716	0.076	ns
	B	2	1650.066	2.649	0.081	ns
	C	2	4649.298	7.464	0.002^**^	
	D	2	920.243	1.477	0.238	ns
At 3-month discharge	A	2	1428.810	2.019	0.144	ns
	B	2	539.570	0.762	0.472	ns
	C	2	2411.682	3.407	0.041^*^	
	D	2	1022.464	1.445	0.246	ns

SSEQ, Stroke Self-Efficacy Questionnaire; DF, degree of freedom; MS, mean square;

* significant, 0.01 < p < 0.05;

** very significant, 0.001 < p < 0.01;

*** highly significant, p < 0.001; non-significance is indicated as ns (p > 0.05).

**Table 10:** ANOVA of the factors affecting the quality of life.

**SS-QoL**	**Factors**	**DF**	**MS**	***F*-value**	***p*-value**	**Significance**
At 1-month discharge	A	2	2488.094	3.811	0.029^*^	
	B	2	3639.984	5.575	0.007^**^	
	C	2	7047.344	10.794	< 0.001^***^	
	D	2	170.365	0.261	0.771	ns
At 3-month discharge	A	2	2000.716	2.593	0.085	ns
	B	2	3011.550	3.903	0.027^*^	
	C	2	3293.438	4.269	0.020^*^	
	D	2	1207.617	1.565	0.220	ns

SS-QoL, Stroke-Specific Quality of Life Scale; DF, degree of freedom; MS, mean square;

* significant, 0.01 < p < 0.05;

** very significant, 0.001 < p < 0.01;

*** *highly significant, p < 0.001; non-significance is indicated as ns (p > 0.05)*.

**Table 11:** ANOVA of the factors affecting social participation.

**IPA**	**Factors**	**DF**	**MS**	***F*-value**	***p*-value**	**Significance**
At 1-month discharge	A	2	1201.317	2.341	0.107	ns
	B	2	2428.542	4.732	0.013^*^	
	C	2	3088.624	6.019	0.006^**^	
	D	2	249.399	0.486	0.618	ns
At 3-month discharge	A	2	1180.424	2.104	0.133	ns
	B	2	1572.176	2.802	0.071	ns
	C	2	2121.134	3.781	0.030^*^	
	D	2	606.379	1.081	0.347	ns

IPA, Impact on Participation and Autonomy questionnaire; DF, degree of freedom; MS, mean square;

* significant, 0.01 < p < 0.05;

** very significant, 0.001 < p < 0.01;

*** highly significant, p < 0.001; non-significance is indicated as ns (p > 0.05).

**Table 12:** ANOVA of the factors affecting the degree of disability.

**mRS**	**Factors**	**DF**	**MS**	***F*-value**	***p*-value**	**Significance**
At discharge	A	2	0.405	0.385	0.683	ns
	B	2	3.395	3.223	0.049^*^	
	C	2	2.795	2.652	0.081	ns
	D	2	0.097	0.092	0.912	ns
At 1-month discharge	A	2	1.630	2.122	0.131	ns
	B	2	3.735	4.861	0.012^*^	
	C	2	3.152	4.102	0.023^*^	
	D	2	0.264	0.343	0.711	ns
At 3-month discharge	A	2	1.821	2.426	0.099	ns
	B	2	2.930	3.904	0.027^*^	
	C	2	1.935	2.578	0.086	ns
	D	2	0.226	0.301	0.742	ns

mRS, modified Rankin Scale; DF, degree of freedom; MS, mean square;

* significant, 0.01 < p < 0.05;

** very significant, 0.001 < p < 0.01;

***highly significant, p < 0.001; non-significance is indicated as ns (p > 0.05).

### Adverse Effects

Among the 57 patients, only one patient accidentally fell during a rehabilitation exercise at home 2 months after discharge, which caused a fracture of the right clavicle. No adverse events occurred in the remaining patients.

## Discussion

In previous studies, researchers have predominantly used randomized controlled methods to assess the advantages of a certain early mobilization program, and have often failed to consider all the factors that affect early mobilization. Therefore, the optimal program for early mobilization is unknown. The MOST framework provides a multiphase strategy to optimize an intervention program. Based on our previous work, we investigated the impact on stroke patients of activity plans with different intensities, frequencies, and durations. Considering the large sample size of the factorial design, we used an orthogonal design to explore the optimal program for early mobilization. Through an orthogonal design table, different intervention programs can be compared, and the most optimal program is finally selected. With this strategy, we addressed the important clinical question of the extent to which early activities influence subsequent rehabilitation decisions. The results of the present study show that the optimal initiation time of early patient activities is 24–48 h and recommend that maximum-intensity activities be undertaken when the ability of the patient allows, including bed and chair transfer, sitting out of bed, standing, and walking or climbing stairs. The duration of each activity can be determined according to the actual situation of the patient, considering that fatigue should be avoided.

This study shows that mobilization at 24–48 h after stroke is more beneficial than mobilization outside this period because it reduces the fatigue experienced by the patients. Krakauer et al. (34) also considered that mobilization of the affected limbs too early after brain injury may hamper brain plasticity because it can weaken γ-aminobutyric acid (GABA-mediated tonic inhibition). Reducing GABA-mediated inhibition in the first few days after the onset of stroke may enlarge the infarct size (34). In the present study, we also found that the intensity of an activity affected early mobilization, and a higher intensity was recommended if the patient's capacity for that activity allowed, because it improved the patient's capacity for daily living, quality of life, and social participation. These results are similar to the results of a randomized controlled study by Tong et al. (22), which had only one outcome measure (mRS score). In the study of Tong at al., “higher intensity” referred to early (24–48 h) intensive mobilization, not just the content of the mobilization, and patients commenced out-of-bed mobilization at ≥ 3 h/day. However, in our study, we actually separated the mobilization dose, and studied the intensity and duration of each activity as two different factors in order to examine the main variables that affect early mobilization.

In this study, the duration of each activity had no impact on early mobilization. A possible reason is that during the implementation of the study, some patients complained of dizziness, fatigue, chest tightness, or other uncomfortable symptoms when they were active for a long time, and they therefore did not comply with the established program. Consequently, the schedule of duration can be based on the actual situation of the patient, to ensure the patient's rehabilitation while reducing the incidence of activity-related intolerance.

In the present study, we found that the appropriate frequency of mobilization could be undertaken in the acute phase of stroke, and 2–3 times a day appeared most beneficial. This differs from the recommendation of Bernhardt and his team (14) that patients suffering acute stroke should undertake low-dose, high-frequency mobilization. The implementation of high-frequency mobilization may increase the patient's fatigue and affect the recovery of their self-efficacy, which will negatively affect their social participation. Our study also showed that factor C (frequency) was the most important factor affecting the early mobilization of patients in terms of indicators BI, SSEQ, SS-QoL, and IPA. However, factor B (intensity) had a stronger effect on mRS than factor C (frequency). Therefore, our results could not conclusively rank the four factors, so further investigation is required in the future.

This project provides innovations in several critically important areas for the future study of early mobilization. First, our project is the first to optimize the early mobilization program for patients with acute ischemic stroke. In it, we examined multiple short- and long-term outcomes. We also based our work on the MOST framework, which is designed specifically to optimize multicomponent treatment programs, but has not previously been applied to the optimization of early mobilization.

Our design comes with several important limitations. Because only a small number of patients are admitted to the stroke unit of this hospital within 24 h of the onset of stroke, the study participants were admitted within 72 h of the onset of stroke. The time at which mobilization was initiated was not included as an influencing factor in the orthogonal design plan. On the basis of three factors and three levels, the time of initiation was included as an independent factor in the data analysis, but the results might be biased.

In the present study, the patients recruited were not representative of the whole stroke population because patients with moderate-to-severe acute ischemic stroke (with an NIHSS score ≥ 16) were excluded. Because our study was conducted at a single center, we recruited insufficient patients to ensure statistical validity (only seven repeated trials of nine programs were performed). When a patient had to be excluded for any reason, we did not include a new patient to complete the experiment. Considering that the purpose of orthogonal design in this study is to weigh the pros and cons, and to combine all evaluation indicators to arrive at the optimal activity plan. No intention-to-treat analysis was performed on the dropped samples, which is also one of the limitations of this study. However, the relatively small sample size and the single-center context of this study still allowed us to draw a meaningful conclusion and to develop an optimized program. This program recommends that high-intensity, moderate-frequency early mobilization at 24–48 h after the onset of stroke is beneficial for these patients. A multicenter study will be undertaken in the future, in which the research subjects will be patients within 24 h of the onset of stroke. The initiation time will be included as an influencing factors as part of a four-factor three-level orthogonal design plan, and the number of repetitions for each combination will be increased to improve the accuracy of the tests and reduce any bias. A future randomized, controlled, multicenter study with a larger sample size can also be used to verify the effectiveness of the optimized program.

## Data Availability Statement

The original contributions presented in the study are included in the article/supplementary material, further inquiries can be directed to the corresponding author/s.

## Ethics Statement

The studies involving human participants were reviewed and approved by Medical Ethics Committee of Drum Tower Hospital affiliated to Nanjing University Medical School. The patients/participants provided their written informed consent to participate in this study.

## Author Contributions

MZ, HS, TP, MW, and YJ performed the study. MZ analyzed data and prepared the manuscript. MZ and QW designed the study and revised the manuscript. All authors contributed to the article and approved the submitted version.

## Conflict of Interest

The authors declare that the research was conducted in the absence of any commercial or financial relationships that could be construed as a potential conflict of interest.
